# The Complex Network of Cytokines and Chemokines in Pediatric Patients with Long-Standing Type 1 Diabetes

**DOI:** 10.3390/ijms25031565

**Published:** 2024-01-26

**Authors:** Anna Wołoszyn-Durkiewicz, Dorota Iwaszkiewicz-Grześ, Dominik Świętoń, Mariusz J. Kujawa, Anna Jankowska, Agata Durawa, Paulina Glasner, Piotr Trzonkowski, Leopold Glasner, Edyta Szurowska, Małgorzata Myśliwiec

**Affiliations:** 1 Department of Pediatrics, Diabetology and Endocrinology, Medical University of Gdańsk, 80-211 Gdańsk, Poland; mysliwiec@gumed.edu.pl; 2 Department of Medical Immunology, Medical University of Gdańsk, 80-211 Gdańsk, Poland; dorota.iwaszkiewicz@gumed.edu.pl (D.I.-G.); ptrzon@gumed.edu.pl (P.T.); 3 2nd Department of Radiology, Medical University of Gdańsk, 80-211 Gdańsk, Poland; dominikswieton@gmail.com (D.Ś.); akfjankowska@gmail.com (A.J.); agata.durawa@gumed.edu.pl (A.D.); edyta.szurowska@gumed.edu.pl (E.S.); 4 Department of Ophthalmology, Medical University of Gdańsk, 80-214 Gdańsk, Poland; paulina.glasner@gumed.edu.pl (P.G.); leopold.glasner@gumed.edu.pl (L.G.); 5 Department of Anesthesiology and Intensive Care, Medical University of Gdańsk, 80-214 Gdańsk, Poland

**Keywords:** type 1 diabetes, cytokines, diabetes angiopathies, atherosclerosis, diabetic retinopathy

## Abstract

Type 1 diabetes (T1D) is a progressive disorder leading to the development of microangiopathies and macroangiopathies. Numerous cytokines and chemokines are involved in the pathogenesis of T1D complications. The study aimed to assess the presence of complications in patients with long-standing T1D and its relationship with serum biomarker concentrations. We examined 52 T1D subjects, with a disease duration ≥4 years and 39 healthy controls. The group of T1D patients was further divided into subgroups based on the duration of the disease (<7 years and ≥7 years) and the metabolic control assessed by the HbAlc level (<8% and ≥8%). We used Luminex Technology to assess a wide range of biomarker concentrations. A 24 h urine test was done to evaluate the rate of albuminuria. Optical coherence tomography (OCT) was conducted to detect early retinopathic changes. Subclinical atherosclerosis was assessed by measuring the carotid intima–media thickness (IMT). T1D patients showed remarkably higher concentrations of EGF, eotaxin/CCL11, MDC/CCL22, sCD40L, TGF-α, and TNF-α. Moreover, we reported statistically significant correlations between cytokines and IMT. Biomarker concentrations depend on numerous factors such as disease duration, metabolic control, and the presence of complications. Although the majority of pediatric T1D patients do not present signs of overt complications, it is indispensable to conduct the screening for angiopathies already in childhood, as its early recognition may attenuate the further progression of complications.

## 1. Introduction

Type 1 diabetes (T1D) is a chronic, progressive autoimmune disorder characterized by the destruction of pancreatic β cells. The etiology of T1D is complex and comprises genetic susceptibility, environmental factors, and immunological mechanisms [1]. A wide range of cytokines and chemokines occurs in T1D pathogenesis [2,3], as well as in the development of diabetic complications [4]. There is abundant literature concerning the impact of tumor necrosis factor-alpha (TNF-α), vascular endothelial growth factor (VEGF), interferon-gamma (IFN-γ), interleukin 1 (IL-1), IL-6, or IL-12 on the destruction of pancreatic β-cells and the development of microangiopathies and macroangiopathies [5,6,7,8]. Although clinically overt complications are rare in children, the first vascular changes may appear even several years after the recognition of T1D. Despite the decline in the incidence rate of angiopathies observed in many countries, complications remain the major factor responsible for death in young T1D patients [9]. Therefore, it is essential to carry out the screening for angiopathies in the T1D pediatric population and prevent its progression within patients. The earliest vascular abnormalities called “endothelial perturbation” may still be reversible. Endothelial perturbation seems to be correlated with the inflammation response, which can be monitored with the use of cytokine concentration measurements [10]. Inflammatory markers may constitute a potential therapeutic target as well [11]. Thus, researchers put a tremendous effort into investigating early biomarkers of T1D complications.

Studies showed that hyperglycemia causes biochemical, structural, and functional abnormalities of vascular cells. Moreover, oxidative stress, hyperosmolarity, the impairment of vascular repair and regeneration, and numerous inflammation pathways may contribute to microcirculation disturbances [12]. Nevertheless, the precise mechanisms of inflammatory responses that occur in the development of T1D complications are only partially known. The role of novel biomarkers is roughly elucidated. Certain cytokines, such as IL-9, IL-17, and IL-33, that may be involved in endothelial dysfunction remain poorly described. IL-9 and IL-17, which are produced by recently discovered T helper 9 (Th-9) and 17 (Th-17) cells, seem to play a crucial role in the initiation of T1D, as well as at the late stage of the disease [13]. Moreover, numerous chemokines and growth factors seem to be responsible for the appearance of vascular disturbances. For instance, researchers proved that the dysregulation of the monocyte chemoattractant protein-1 (MCP-1) level may decrease the number of endothelial progenitor cells (EPC) and attenuate its function. EPCs occur in cardiovascular repair and regeneration. Thus, they have a therapeutic potential in angiopathies [12]. However, the mechanisms of action of other chemokines such as IL-8, eotaxin, or macrophage-derived chemokine (MDC) are unclear. Despite the ample number of studies concerning the impact of VEGF in T1D complications [6,14], there is scant literature on the role of epidermal growth factor (EGF), fibroblast growth factor 2 (FGF-2), or transforming growth factor alpha (TGF-α) in T1D. Moreover, frequently different studies point to contradictory results [15], and certain cytokines may play both pro- and anti-inflammatory roles in T1D. IL-12 is an example of a dual-role cytokine. It is believed to be a protective factor as it inhibits the release of proinflammatory cytokines. However, it also increases the number of CD4^+^ T cells that infiltrate islet cells, and it may accelerate the onset of T1D [2]. Considering the complexity of the T1D complications pathophysiology, there is a high need to deepen the knowledge in this area.

We aimed to assess early angiopathic changes in the long-standing T1D pediatric population and establish their relationship with a wide range of cytokines, chemokines, and growth factor levels, including novel biomarkers such as IL-9 and IL-17. In order to assess microvascular and macrovascular complications, we performed laboratory and imaging tests. We paid particular attention to the presence of dyslipidemia as it constitutes one of the major risk factors for cardiovascular events. Moreover, we investigated the relationship between biomarker concentrations and glycated hemoglobin (HbA1c) levels, the duration of T1D, Vitamin D levels, and total daily doses (TDI) of insulin.

## 2. Results

### 2.1. Clinical Characteristics of Patients

**Figure 1:**
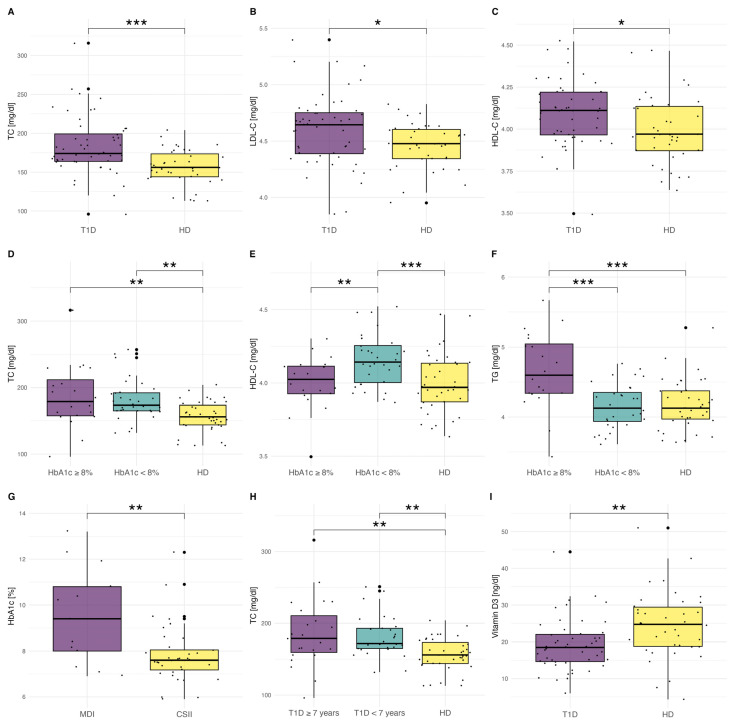
Clinical characteristics of patients. (**A**–**C**): The comparison of TC, LDL-C, and HDL-C levels between the T1D group and healthy individuals. LDL-C and HDL-C were normalized using logarithmic transformation and presented as normally distributed log-transformed data. (**D**–**F**): Level of lipids: TG, HDL-C, and TC in healthy control (*n* = 39) and T1D patients divided into two groups: HbA1c < 8% (n = 32) and HbA1c ≥ 8% (*n* = 20). The results are presented as mean ± SD. HDL-C and TG were normalized using logarithmic transformation and presented as normally distributed log-transformed data. (**G**): The difference in HbA1c level in patients with T1D treated with MDI (*n* = 13) and CSII (*n* = 39) therapy. (**H**): The level of TC in healthy control (*n* = 39) and T1D patients divided into two groups: T1D ≥ 7 years (*n* = 32) and T1D < 7 years (*n* = 20). The differences in LDL-C and HDL-C were not statistically significant. (**I**): The comparison of Vitamin D levels between T1D and HD groups. The results are presented as mean ± SD. Significance was calculated using the ANOVA test with subsequent post-hoc analysis of contrasts. Significant results are marked with * (*p* < 0.05), ** (*p* < 0.01), or *** (*p* < 0.001). Dots show the distribution of data.

The exact characteristic of patients is presented in Table 1, and the most important differences are presented in Figure 1. There were no significant differences in the mean age and BMI between the groups. The mean duration of T1D was 7.1 ± 3.0 years, and the mean age of the disease onset was 6.2 ± 3.1 years. The mean HbA1c in the T1D group was 8.2 ± 1.7% (66.1 mmol/L). The majority of patients (*n* = 39, 75%) were treated with the use of continuous subcutaneous insulin infusion (CSII), whereas the rest (*n* = 13) were treated with multiple daily insulin injections (MDI).

As many as 36.5% (*n* = 19) of T1D patients were diagnosed with dyslipidemia. Patients with T1D presented significantly higher concentrations of total cholesterol (TC), (*p* < 0.001), low-density lipoprotein cholesterol (LDL-C), (*p* = 0.022), and high-density lipoprotein cholesterol (HDL-C), (*p* = 0.013), (Figure 1A–C).

Subsequently, we divided T1D patients into two subgroups depending on HbA1c level, and we compared their lipid profiles to the healthy individuals (control). We observed statistically significant differences. T1D patients with HbA1c ≥ 8% (63.9 mmol/mol) presented the highest level of TC (*p* < 0.001) and triglycerides (TG), (*p* < 0.001) (Figure 1D–F). The LDL-C concentration was also the highest in the group of patients with the worst metabolic control, but the difference was not statistically significant (*p* = 0.071). We reported the highest HDL-C level in T1D patients with HbA1c < 8%, even in comparison to healthy individuals (*p* < 0.001), (Figure 1E).

Moreover, we observed a significantly higher level of HbA1c in the MDI group (9.6 ± 2.1%, 81.4 mmol/L) than in the CSII group (7.8 ± 1.2%, 61.7 mmol/L) (*p* = 0.007), (Figure 1G).

On the other hand, when we divided patients according to T1D duration, we stated only one statistically significant difference in TC concentrations. Individuals affected with T1D ≥ 7 years presented the highest TC (*p* < 0.001), (Figure 1H).

Vitamin D level was significantly lower in T1D patients (*p* = 0.002; Figure 1I) with a mean concentration of 19.2 ± 7.0 ng/dL. The majority of T1D patients had Vitamin D deficiency (*n* = 33, 63.5%). Only five patients (9.6%) had normal Vitamin D levels (>30 ng/dL), and 14 patients presented (26.9%) suboptimal concentration (20–30 ng/dL).

We did not find a statistically significant difference in albumin concentration in urine with mean albuminuria in the T1D group of 12.7 ± 23.7 mg/24 h. Only seven T1D patients (13.5%) were diagnosed with an increased rate of albuminuria above 30 mg/24 h. We did not report any statistically significant differences in mean retinal central subfield and center point thickness in the OCT. Moreover, the patients did not present any abnormalities suggesting diabetic retinopathy.

Mean carotid intima–media thickness (IMT) measurements were practically the same in both groups with mean IMT—0.4 ± 0.1 mm in T1D patients. Only seven (13.5%) patients had IMT above the 95th percentile according to the reference values by Doyon et al. [16].

We did not observe any statistically significant differences in the incidence rate of diabetes (*p* = 0.097), dyslipidemia (*p* = 0.128), thyroid diseases (*p* = 0.581), hypertension (*p* = 0.586), or coronary heart disease (*p* = 0.759) in the family compared to the control group.

Cytokines and chemokines concentrations.

### 2.2. T1D vs. Healthy Individuals

We did not obtain the results of cytokines levels in one patient due to a laboratory error. As we obtained an extensive amount of data, we decided to present only statistically significant results.

**Figure 2:**
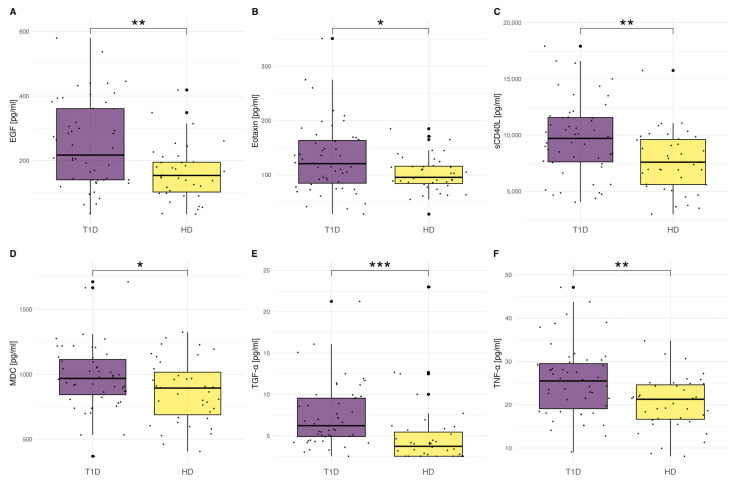
(**A**–**F**) The comparison of cytokines, chemokines, and growth factor levels between the T1D patients (*n* = 51) and the control group (*n* = 39). The significance was calculated using ANOVA with subsequent post-hoc analysis of contrasts. In the case of the TGF-α level, the Kruskal–Wallis test with post-hoc Wilcoxon test was used due to the lack of normal distribution of variables. Significant results are marked with * (*p* < 0.05), ** (*p* < 0.01), or *** (*p* < 0.001). Dots show the distribution of data.

We noticed that T1D patients showed remarkably higher concentrations of proinflammatory TGF-α (*p* < 0.001), TNF-α (*p* = 0.002), EGF (*p* < 0.001), eotaxin (*p* = 0.017), MDC (*p* = 0.036), and sCD40L (*p* = 0.002), which are presented in Figure 2A–F and Table 2. We also noticed a trend towards significance in IP-10/CXCL10 (*p* = 0.059). The differences in other cytokines levels were not statistically significant.

### 2.3. Duration of the Disease

We did not observe correlations between cytokines, chemokines, growth factors, and the duration of the disease. The differences in biomarker levels between individuals affected with T1D ≥ 7 years (*n* = 23) and <7 years (*n* = 28) were not statistically significant.

However, we reported correlations between the duration of the disease and HbA1c level (r = 0.290, *p* = 0.040), (Figure S1A) and the TG level (r = 0.29, *p* = 0.041) (Figure S1B).

### 2.4. TDI

We reported statistically significant negative correlations between FGF-2 (r = −0.380, *p* = 0.008), GM-CSF (r = −0.400, *p* = 0.004), fractalkine (r = −0.330, *p* = 0.021), INF-α2 (r = −0.340, *p* = 0.016), IFN-γ (r = −0.360, *p* = 0.011), IL-17 (r = −0.43, *p* = 0.023), and the TDI (U/kg) (Supplementary Figure S2A–F).

### 2.5. HbA1c

We found statistically significant positive correlations in T1D group between IL-8/CXCL8 (r = 0.290, *p* = 0.043), MIP-1α/CCL3 (r = 0.480, *p* < 0.001), and HbA1c levels (Supplementary Figure S3A,B).

**Figure 3:**
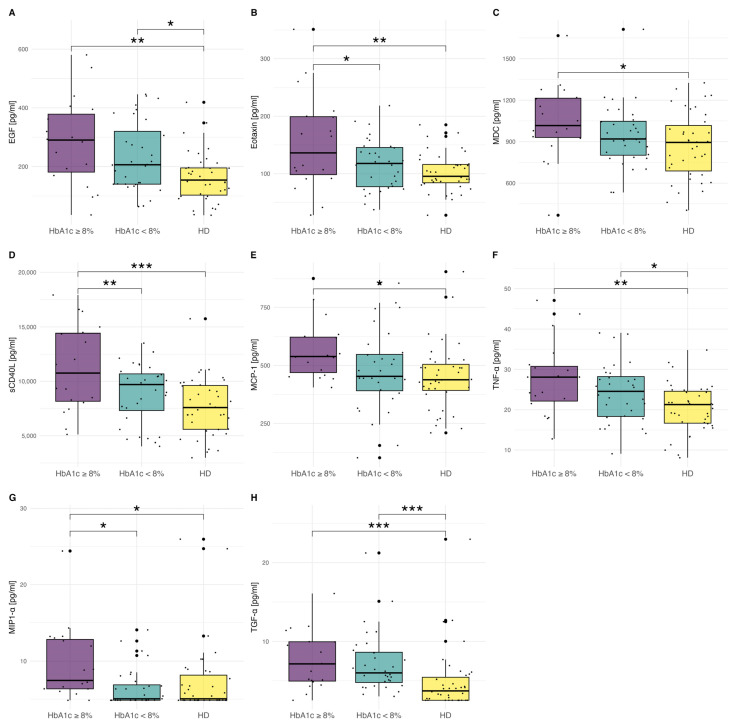
(**A**–**H**) Concentrations of cytokines in T1D patients with HbA1c ≥ 8% (63.9 mmol/L), (*n* = 20), HbA1c < 8% (*n* = 32), and in the HD group. The significance was calculated using ANOVA with subsequent post-hoc analysis of contrasts. The results are presented as mean ± SD. In the case of MIP-1α and TGF-α, the significance was calculated using the Kruskal–Wallis test with the post-hoc Wilcoxon test due to the lack of normal distribution of variables. Significant results are marked with * (*p* < 0.05), ** (*p* < 0.01), or *** (*p* < 0.001). Dots show the distribution of data.

In addition, we reported statistically significant differences in cytokines levels after the division of T1D patients into two subgroups: HbA1c < 8% (63.9 mmol/L), (*n* = 32) and HbA1c ≥ 8% (*n* = 20). Proinflammatory cytokine and chemokine concentrations were the highest in children with HbA1c ≥ 8%. Eotaxin, sCD40L, and MIP-1α/CCL3 concentrations were significantly higher in the group with worse metabolic control (Figure 3A–H, (Table S1)).

### 2.6. Albuminuria

We did not report statistically significant correlations between albumin concentration in urine and cytokines, chemokines, or growth factor concentrations.

### 2.7. IMT

**Figure 4:**
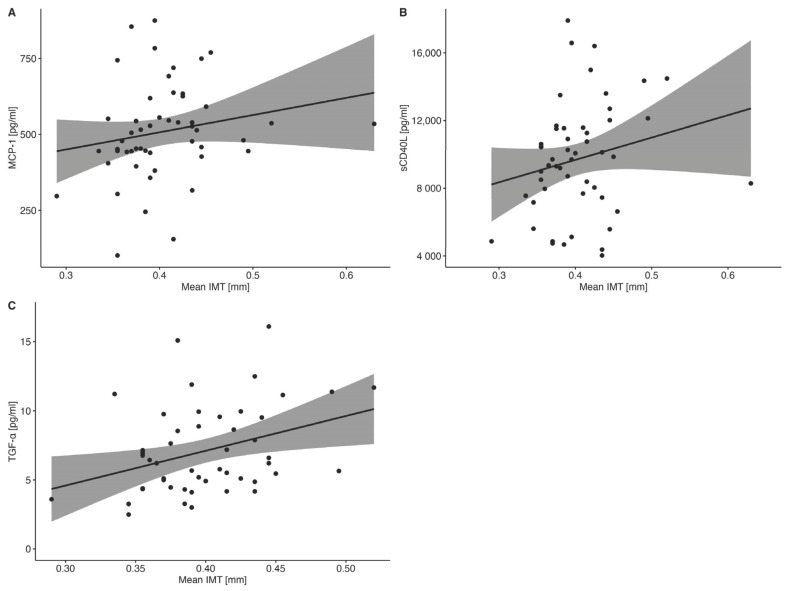
(**A**–**C**). Statistically significant correlations between cytokine concentrations [pg/mL] and mean carotid IMT [mm] in T1D patients. Correlations between measured parameters were analyzed using Spearman’s rank correlation. The grey region denotes the 95% confidence of interval. Dots show the distribution of data.

There were positive statistically significant correlations between MCP-1/CCL2 (r = 0.300, *p* = 0.034), sCD40L (r = 0.290, *p* = 0.044), TGF-α (r = 0.330, *p* = 0.021), and the mean IMT in T1D group (Figure 4A–C). Moreover, we observed a statistically significant correlation between the mean IMT and TC level (Supplementary Figure S4).

### 2.8. OCT

We did not find any statistically significant correlations between mean central subfield thickness (CSF (CSF), central point thickness (CPT) in OCT, and cytokine, chemokine, or growth factor concentrations in the T1D group. The correlation between CSF and CPT was very strong (r = 0.960, *p* < 0.001).

### 2.9. Correlations between Cytokines

We found numerous statistically significant correlations between cytokines. The strongest correlations are listed in Table 3 and presented in Supplementary Figure S6.

## 3. Discussion

In the presented study, we demonstrated that numerous cytokines and chemokines play a crucial role in long-standing T1D and predispose to the development of vascular complications. We reported increased concentrations of proinflammatory cytokines such as EGF, eotaxin/CCL11, MDC/CCL22, sCD40L, TGF-α, and TNF-α in patients with T1D in comparison to healthy individuals.

One of the most important biomarkers occurring in T1D pathogenesis is TNF-α. It is a pleiotropic proinflammatory cytokine that induces a wide variety of inflammatory responses. Over 20 years ago, researchers detected the infiltration of pancreatic islets by TNF-α producing cells [17,18]. It is a key factor involved in the destruction of pancreatic β cells [19,20]. Previous studies showed increased levels of TNF-α in T1D patients, which was consistent with our work [6,21,22,23,24].

Moreover, we demonstrated the increased EGF and sCD40L concentrations in T1D patients. EGF promotes cell proliferation and differentiation. There is a scarce number of studies focusing on its role in T1D [25]. Similarly, other studies showed an increased level of serum EGF in T1D individuals [25,26,27,28].

The sCD40L is a soluble, active form of CD40L. It has both proinflammatory and procoagulant properties [29]. Other studies reported higher CD40L levels in T1D patients, as in our research, and suggested its essential role in the development of microangiopathies [30,31].

The role of other cytokines and chemokines is not fully elucidated. Eotaxin/CCL11 is a chemokine particularly known for eosinophil recruitment. However, its role is more complex [32]. It also affects the surface adhesion molecules of endothelial cells [33]. Hessner et al. found the overexpression of eotaxin/CCL11 in the pancreatic lymph nodes of diabetic rats and proved its involvement in the pathogenesis of T1D [34].

In addition, we found a higher concentration of MDC/CCL22 level in patients affected with T1D. It is a chemokine produced predominantly by macrophages and dendritic cells. Previous studies showed that the blockade of MDC/CCL22 in non-obese diabetic mice decreased insulitis [35].

Moreover, we detected a high concentration of TGF-α in patients with long-standing T1D. It stimulates cell proliferation and migration. It is also involved in angiogenesis [36]. Studies proved that TGF-α is responsible for the progression of diabetic nephropathy in patients with Type 2 diabetes [37]. Its role in Type 1 diabetes is still unclear.

Moreover, we demonstrated an interesting relation between T1D duration and metabolic control of the disease. The HbA1c level tended to increase along with T1D duration. Similarly, Hilliard et al. showed that longer T1D duration was associated with poorer T1D management [38]. Moreover, we found a correlation between the T1D duration and TG level. Previous studies proved that TG concentration is a predictive factor for retinopathy and nephropathy development in T1D patients [39].

Furthermore, we observed numerous correlations between cytokine concentrations and TDI/kg. Surprisingly, all of the correlations were negative. These observations were in contrast to our expectations. We may postulate that in patients, who still have their endogenous insulin production, numerous proinflammatory cytokines, involved in the destruction of pancreatic β cells, are released. As a result, the production of anti-inflammatory cytokines such as IL-4 and IL-10 is enhanced to counteract the effects of proinflammatory factors. On the other hand, many of these cytokines occur in the development and progression of diabetic complications [7,40]. Lower levels of cytokines in patients with higher insulin requirements were probably caused by the fact that the majority of our patients had not developed even early stages of complications yet. Moreover, the other possible explanation of negative correlations between TDI/kg and cytokines levels is the fact that insulin has pleiotropic anti-inflammatory properties. It modulates inflammatory mediators and exerts the vasodilatory effects [41]. This finding warrants further investigations.

In our study, MDI patients presented worse metabolic control in comparison to the CSII group. This finding was in agreement with a large cohort study [42]. We stated a positive correlation between IL-8/CXCL8, MIP-1α/CCL3, and HbA1c levels. Similarly, previous studies outlined the association between serum IL-8 [43] and MIP-1α/CCL3 [4] concentrations and HbA1c levels. Additionally, we observed statistically higher eotaxin, sCD40L, and MIP-1α/CCL3 concentrations in the group of T1D patients with HbA1c ≥ 8% in comparison to T1D individuals with HbA1c < 8%. It seems that the worse metabolic control may induce an increase in proinflammatory biomarkers levels. The available literature points to the role of MIP-1α/CCL3 in T1D [4]. However, the data on its relation to metabolic control are lacking. MIP-1α/CCL3 is a potent chemoattractant responsible for the recruitment of proinflammatory cytokines, and its level is elevated in T1D subjects [4].

Moreover, 36.5% (*n* = 19) of T1D patients were diagnosed with dyslipidemia. We observed statistically higher TC, LDL-C, and HDL-C levels in T1D subjects. The TC and TG concentrations were the highest in the group of patients with HbA1c ≥ 8% (63.9 mmol/L). Interestingly, we did not find any statistically significant differences between LDL-C and TC levels in the T1D group depending on HbA1c, whereas previous studies underlined the influence of glycemic control on elevated TG and LDL-C concentrations [44,45]. HDL-cholesterol has a beneficial role in T1D as it expresses antioxidant and anti-inflammatory roles. Patients with optimal metabolic control often present normal or even high HDL-C levels. However, the cardiovascular risk remains elevated. This phenomenon may be explained by the fact that HDL-C protein composition is altered in T1D patients, and its protective capacities may be compromised [44,46]. Clinicians should pay attention to the screening for dyslipidemia as it is one of the major risk factors for cardiovascular complications.

Cytokines play a pivotal role in the development and progression of diabetic complications. It is common knowledge that a wide range of biomarkers is involved in the development of diabetic nephropathy [47]. Surprisingly, we did not report any statistically significant correlations between biomarker levels and albumin concentration in urine. We can assume that it was caused by a small number of patients with increased albuminuria.

The role of several cytokines such as IL-6, IL-8, MCP-1/CCL2, and VEGF is well-known in diabetic retinopathy (DR) [48]. Moreover, anti-VEGF agents are applied for DR therapy [49]. Researchers are still seeking new agents involved in the pathogenesis of DR. Moreover, novel imaging methods such as OCT are used to determine the changes in the retinal vasculature. OCT is a non-invasive tool enabling the detection of DR abnormalities [50]. However, pediatric norms do not exist, and fluorescein angiography is common in clinical practice. In our study, we showed similar CPT and CSF in the group of T1D and healthy patients. This result was in agreement with a study performed on adults by Bressler et al., who showed similar CSF values in the diabetic and control groups [51]. Mastropasqua et al. demonstrated positive correlations between central macular thickness and GRO/CXCL1, VEGF, fractalkine, IP-10/CXCL10, and IL-12p70 in aqueous humor in subjects with diabetic macular edema [52]. Both IL-12, as an anti-angiogenic factor, and IL-1Ra seem to have a protective role in DR. Despite the undeniable involvement of biomarkers in the development of DR, we did not report any statistically significant correlations between cytokines and CSF and CPT in OCT. However, it should be emphasized that T1D patients enrolled in the study group have not presented any retinal abnormalities yet.

Even though the clinical manifestation of cardiovascular disease in children is rare, the first macroangiopathic pathologies, such as early atherosclerotic changes, appear already in childhood. Subclinical atherosclerosis may be determined with the use of carotid IMT. Studies showed ambiguous results concerning the difference in IMT between T1D and healthy patients [53]. We did not report any statistically significant difference between the two groups. However, seven patients (13.5%) already presented increased IMT (>95th percentile according to the Doyon et al. norms) [16]. We found that MCP-1/CCL2, sCD40L, and TGF-α were positively correlated with the mean carotid IMT in the T1D group. The role of MCP-1/CCL2 and sCD40L in atherosclerosis is well-established [54]. TGF-α also seems to be involved in atherosclerosis as an angiogenic factor [55].

Moreover, we would like to highlight the important role of Vitamin D in T1D. Despite the Vitamin D deficiency in the majority of our patients, we did not find any statistically significant correlations between Vitamin D and cytokine levels. Previous studies confirmed the association between proinflammatory cytokines and deficient Vitamin D concentrations in the T1D group [56]. It should be emphasized that Vitamin D deficiency contributes to T1D pathogenesis and the development of diabetic complications [57,58].

Finally, we observed numerous correlations between cytokines concentrations. Some interactions between biomarkers are well-known like the inhibition of IL-1α and β signaling by IL-1Ra [59] or the induction of MCP-1 by GM-CSF [60]. However, the complex cytokines network remains unclear to a large extent. Our results pointing to an interaction between GM-CSF and MCP-1 are consistent with research by Tanimoto et al. [60]. A previous study reported the enhancement of fractalkine by G-CSF in adults with Type 2 diabetes [61]. We have not found any data explaining the interaction between EGF and sCD40L or INF-α2 and GM-CSF.

To our knowledge, it is the first research to investigate the relationship between a wide range of cytokines, chemokines, and growth factors examined with Luminex technology, and IMT or OCT parameters in the pediatric T1D population, which is certainly the novelty of our study. However, we are aware of the fact that our study has a few limitations. The majority of patients did not present any complications yet. Therefore, we should consider the repetition of our study over several years to check the progression of vascular changes and differences in cytokine concentrations. Moreover, HbA1c, as a metabolic control parameter, is not as good as new parameters such as time in range (TIR) and glycemic variability (GV). The majority of our patients did not use continuous glucose monitoring (CGM). Nevertheless, thanks to the increasing access to CGM, we plan to investigate the correlations between TIR, GV, and cytokine concentrations in the future.

## 4. Materials and Methods

### 4.1. Study Group

**Figure 5:**
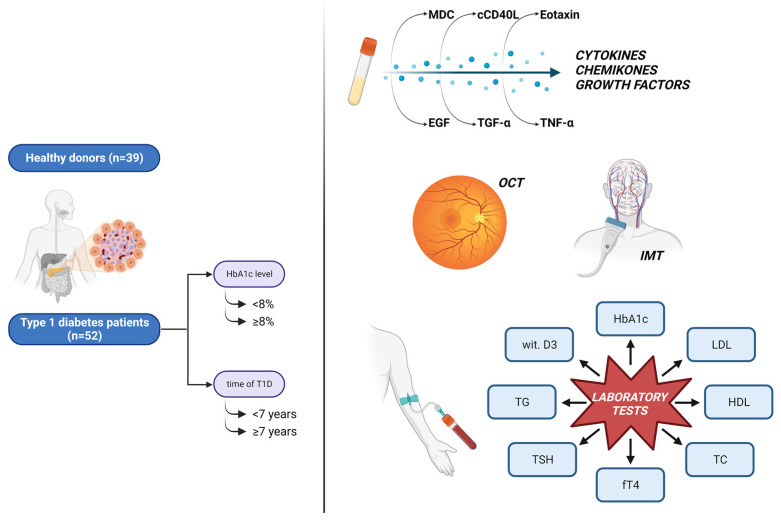
The flowchart presents the division into the subgroups and the tests conducted on the patients.

The whole study group consisted of 52 subjects affected with T1D and 39 healthy volunteers (control) matched by age and gender. The group of T1D patients was further divided into subgroups based on the duration of the disease (<7 years and ≥7 years) and metabolic control assessed by the HbAlc level (<8% and ≥8%) (Figure 5). In the study we included patients who were under the care of the Clinic of Pediatrics, Diabetology, and Endocrinology at the University Clinical Center in Gdansk, Poland. Inclusion criteria were those who were diagnosed with T1D according to the International Society for Pediatric and Adolescent Diabetes criteria [62], aged 8–18 years and had a diabetes duration of more than four years. Exclusion criteria included diabetic ketoacidosis at the moment of recruitment to the study, ongoing infection, uncompensated celiac disease, hypothyroidism or other endocrine disorders, chronic respiratory, and renal diseases, as well as neurological and immunological disorders.

We set cut-off points of 8% for HbA1c and 7 years of T1D duration, as they were close to median values in the cohort of patients from the diabetes center in Gdansk, who fulfilled the inclusion criteria.

The control group consisted of healthy children without chronic diseases and long-term treatment. They were recruited from schools or were our former patients who were referred to our Clinic due to the suspicion of endocrine disorders, which were finally excluded (for example, pre-laboratory and laboratory mistakes or the incorrect interpretation of diagnostics).

All of the patients were in full health. The active infection was excluded on the basis of a medical interview regarding the symptoms of infection during the last 7 days, precise physical examination, and laboratory tests (complete blood count and C-reactive protein).

Additionally, we gathered information on family history of diabetes, thyroid diseases, dyslipidemia, coronary heart diseases, and hypertension. We considered the health history of three generations. We based it on verbal information from parents.

The puberty was assessed with the use of the Tanner scale (1–5). BMI percentile was evaluated using OLAF percentile charts for the Polish pediatric population [63].

The study was conducted according to the guidelines of the Declaration of Helsinki and approved by the independent bioethics committee for scientific research at the Medical University in Gdansk, Poland, No 565/2018. We obtained informed written consent from all of the patients and their parents or legal guardians before enrolment into the study.

All of the tests conducted on the T1D patients and healthy individuals are presented in Figure 5.

### 4.2. Serum Sample Collection

We collected fasting serum samples from all of the participants (the T1D and the control groups). We centrifuged blood samples at 1000 rpm for 10 min within 30 min after blood collection. The serum of each patient was separated into three sterile 1.5 mL Eppendorf tubes and frozen at −80 °C until used, but not longer than 12 months. Samples were thawed at room temperature and immediately used in Cytokine/Chemokine Array analysis.

### 4.3. Human Cytokine/Chemokine Magnetic Bead Panel

We used a Milliplex MAP Human Cytokine/Chemokine Magnetic Bead Panel (HCYTMAG-60K-PX38, Merck Millipore, St. Charles, MO, USA) to assess the broad spectrum of cytokine and chemokine concentrations according to the manufacturer’s instruction. The kit protocol is available online [64]. Briefly, Luminex technology enables the establishment of immunoassays on the surface of fluorescent-coded magnetic beads—MagPlex^®^-C microspheres. Multiplex assays allow simultaneous detection of multiple analytes in a single sample. This is particularly advantageous when working with a large number of samples or when testing multiple cytokines or chemokines. It can significantly improve the efficiency of data generation compared to testing each target individually. If the goal of the study is to obtain a comprehensive overview of the immune response or inflammatory profile, multiplexing techniques can be used to measure multiple analytes simultaneously. This can provide a more comprehensive understanding of the biological system under study.

The multiplex assay consisted of 38 analytes: soluble CD40 ligand (sCD40L), EGF, eotaxin, also known as C-C motif chemokine 11 (CCL11), FGF-2, fms-like tyrosine kinase 3 (Flt-3 ligand), fractalkine, granulocyte colony-stimulating factor (G-CSF), granulocyte-macrophage colony-stimulating factor (GM-CSF), growth-regulating oncogene (GRO), also known as C-X-C Motif Chemokine Ligand 1 (CXCL1), interferon alpha-2 (IFN-α2), IFN-γ, IL-1α, IL-1β, Il-1 receptor antagonist (IL-1Ra), IL-2, IL-3, IL-4, IL-5, IL-6, IL-7, IL-8/C-X-C ligand motif 8 (CXCL8), IL-9, IL-10, IL-12p40, IL-12p70, IL-13, IL-15, IL-17, interferon gamma-induced protein 10 (IP-10)/C-X-C motif chemokine ligand 10 (CXCL10), *MCP*-1/C-C motif ligand 2 (CCL2), MCP-3/C-C-motif ligand 7 (CCL7), MDC/C-C motif ligand 22 (CCL22), macrophage inflammatory protein-1 alpha (MIP-1α)/C-C-motif ligand 3 (CCL3), macrophage inflammatory protein 1 beta (MIP-1β)/C-C motif ligand 4 (CCL4), TGF-α, TNF-α, tumor necrosis factor beta (TNF-β), and VEGF. Each biomarker level was measured in triplicate. The assay sensitivities of cytokines and chemokines ranged from 0.7 to 26.3 pg/mL, while the precision varied from 1.5 to 7.2% of the intra-assay coefficient of variation.

### 4.4. Laboratory Tests

Biochemical tests, including complete blood count, C-reactive protein, electrolytes, lipid profile, renal, and hepatic function markers, as well as immunological screening for concomitant diseases (autoimmune thyroiditis and celiac disease, for example), were analyzed. Moreover, we performed 24 h urine collection to assess albumin concentration. The metabolic control in T1D subjects was based on HbA1c level. We divided patients with diabetes into two subgroups (Group 1 with HbA1c < 8% (*n* = 32) and Group 2 (*n* = 20) with HbA1c ≥ 8%) to compare the differences in cytokines levels. Moreover, we created two subgroups according to the duration of diabetes—<7 years (*n* = 28) and ≥7 years (*n* = 23). All of the laboratory tests were conducted in the certified Central Laboratory at the University Clinical Center in Gdansk.

### 4.5. Optical Coherence Tomography (OCT)

We assessed early retinal changes in optical coherence tomography (OCT) using Triton Plus (Topcon, Tokyo, Japan). The manufacturer’s manual is available online [65]. The eyes were scanned, providing high-resolution 3D images of retinal vasculature. We measured CPT and CSF obtained from early-treatment diabetic retinopathy study (ETDRS) subfields. CPT was determined as an average thickness calculated automatically at the intersection of six radial scans. The mean CSF and CPT values were obtained by averaging the left and right sides.

### 4.6. Carotid Intima–Media Thickness (IMT)

The ultrasound was performed with Philips Epiq 5 (Philips Ultrasound, Bothell, WA, USA) using linear, high-frequency probes 12–18 MHz. The examination was conducted after 5 minutes of rest. The patient’s head was located in the axis of the body and turned to the opposite direction of the examined artery, at about 45 degrees. The transducer was placed perpendicularly to the common carotid artery. Common artery IMT complex was measured around 1.5–2 cm below the carotid bifurcation. The measurements were recorded in the end-diastolic phase and repeated three times for each site. Subsequently, we calculated the arithmetic mean from the received results and averaged the results from the right and left sides.

### 4.7. Statistical Analysis

All statistical analyses were performed in R version 4.0.4 (Vienna, Austria) [66]. The following R packages were used for statistical analyses: dplyr, tidyverse, ggplot, ggpubr, stats, emmeans, and lmtest. The normal distribution of experimental data was verified using the Shapiro–Wilk W test. Normally distributed data were presented as mean ± standard deviation (SD). Non-normally distributed data were first normalized using logarithmic transformation, and if the normalization was successful, normally distributed log-transformed data were used in further analyses. The Breusch–Pagan test was employed to verify the homoscedasticity of data. Outliers, defined as values more than 3 SDs from the average, were removed from further analyses.

In the case of normally distributed data, all between-group comparisons, i.e., comparisons based on the presence/absence of T1D (healthy versus T1D), the duration of T1D (healthy controls/<7 years/≥7 years), and the T1D metabolic control (healthy controls/<8% HbA_1C_/≥8% HbA_1C_) were analyzed using analysis of variance (ANOVA) with subsequent post-hoc analysis of contrasts. Multiple comparisons were accounted for by employing the Benjamini and Hochberg FDR correction [67].

Data that failed to show normal distribution even following the log transformation are presented as medians with corresponding interquartile ranges (IQR). To analyze the above-mentioned between-group comparisons, three separate Kruskal–Wallis H test-based analyses, with resultant main effect levels of significance being adjusted for multiple comparisons using the Benjamini and Hochberg FDR correction, were performed. FDR-adjusted Wilcoxon test was then used to analyze the multiple comparisons in the post-hoc analysis.

Simple correlations between measured parameters were analyzed using Spearman’s rank correlation and are expressed using the Spearman correlation coefficient (r_SP_). Only statistically significant correlations with |r_SP_| > 0.5 are shown. Statistical significance was inferred for *p* < 0.05.

## 5. Conclusions

The results of our study shed light on the important role of cytokines and chemokines in long-standing T1D and the development of diabetic complications. We may assume that biomarker concentrations depend on numerous factors such as disease duration, metabolic control, and the presence of vascular changes. Although the majority of pediatric T1D patients do not present signs of overt complications, it is indispensable to conduct the screening for both microangiopathies and macroangiopathies already in childhood. Moreover, clinicians should conduct regular lipid profile control and recommend Vitamin D supplementation in patients with T1D. Early recognition of first vascular changes is crucial to attenuate the progression of angiopathies. Moreover, it is worth considering the implementation of potential therapeutic strategies for diabetic complications. Further investigations are required to fully understand the underlying mechanisms of action of cytokines and chemokines in diabetes.

## Figures and Tables

**Table 1:** Baseline clinical characteristics of the patients. Data are presented as mean values ± SD. * Non-normally distributed data were normalized using logarithmic transformation and presented as normally distributed log-transformed data. ** Data that failed to show normal distribution even following the log transformation are presented as medians with corresponding interquartile ranges (IQR).

Characteristics	T1D Group (*n* = 52)	HD Group (*n* = 39)	*p*-Value
Male; *n* (%)	26 (50%)	20 (51.3%)	0.904
Mean age ± SD (years)	13.8 ± 2.9	13.5 ± 2.4	0.628
Tanner scale, *n* (%)			0.478
1	10 (19.2)	6 (15.4)	
2	7 (13.5)	7 (17.9)	
3	9 (17.3)	8 (20.5)	
4	9 (17.3)	10 (25.6)	
5	17 (32.7)	7 (17.9)	
BMI (kg/m^2^)	20.9 ± 3.5	19.9 ± 2.4	0.214
BMI (percentile)	63.1 ± 20.6	59.7 ± 25.4	0.591
Duration of diabetes (years)	7.1 ± 3.0		
Age at the onset of diabetes (years)	6.2 ± 3.1		
CSII; *n* (%)	39 (75.0)		
MDI; *n* (%)	13 (25.0)		
CSII duration (years)	7.1 ± 3.0		
The total daily dose of insulin (U/kg)	0.8 ± 0.2		
Mean C-peptide (ng/mL)	0.3 ± 0.1		
Mean HbA1c (%)	8.2 ± 1.7	5.2 ± 1.5	<0.001
Dyslipidemia, *n* (%)	19 (36.5)	3 (7.7)	0.001
TC (mg/dL)	183.2 ± 37.6	156.6 ± 22.5	<0.001
LDL-C (mg/dL) *	104.4 ± 34.4	88.4 ± 16.9	0.022
HDL-C (mg/dL) *	61.3 ± 11.5	55.3 ± 11.5	0.013
TG (mg/dL) *	87.5 ± 50.3	69.8 ± 29.1	0.071
Vitamin D (ng/dL)	19.2 ± 7.0	24.5 ± 9.2	0.002
CPT mean (µm)	209.1 ± 25.6	209.2 ± 16.5	0.998
CSF mean (µm)	237.7 ± 19.8	233.9 ± 19.9	0.613
Mean IMT (mm)	0.4 ± 0.1	0.4 ± 0.0	0.248
**Family history**			
Diabetes, *n* (%)	38 (73.1)	22 (56.4)	0.097
Thyroid diseases, *n* (%)	29 (55.8)	24 (61.5)	0.581
Dyslipidemia, *n* (%)	18 (35.3)	20 (51.3)	0.128
Coronary heart disease, *n* (%)	21 (40.4)	17 (43.6)	0.759
Hypertension *n* (%)	27 (51.9)	18 (46.2)	0.586
**Characteristics**	**T1D group** (*n* **= 52**) median/IQR	**HD group **(*n*** = 39**) median/IQR	*p*
Albuminuria (mg/24 h) **	4.1 (17.8)	6.5 (9.9)	0.723

Abbreviations: CSII—continuous subcutaneous insulin infusion, MDI—multiple dose injections, TC—total cholesterol, LDL-C—low lipoprotein cholesterol, HDL-C high—density lipoprotein cholesterol, TG—triglycerides, CPT—central point thickness, CSF—central subfield thickness, IMT—intima-media thickness.

**Table 2:** Cytokine and chemokine concentrations in T1D patients in comparison to the HD group. Data are presented as mean values ± SD. F and q values were reported in the case of the ANOVA test. ˟ Data that failed to show normal distribution even following the log transformation were analyzed using the Kruskal–Wallis test with post-hoc Wilcoxon test and are presented as medians with corresponding interquartile ranges (IQR).

Cytokines Concentrations (pg/mL)	T1D Group (*n* = 51)	HD Group (*n* = 39)	*p*-Value	F	q T1D/HD
EGF	252.0 ± 130.5	162.6 ± 83.9	<0.001	13.879	<0.001
AXIN/CCL11	129.3 ± 63.4	102.2 ± 33.0	0.017	5.898	0.017
MDC/CCL22	981.1 ± 246.0	871.2 ± 238.9	0.036	4.521	0.036
sCD40L	9728.8 ± 3343.5	7645.8 ± 2638.8	0.002	10.247	0.002
TNF-α	25.5 ± 8.1	20.4 ± 6.0	0.002	10.781	0.002
˟ Cytokines concentrations (pg/mL)	T1D group (*n* = 51)	HD group (*n* = 39)	*p*-value	Chi-square	
TGF-α	6.21 (4.65)	3.71 (2.93)	<0.001	19.560	

**Table 3:** The strongest correlations between cytokine concentrations in T1D patients.

Cytokines Concentrations (pg/mL)	Cytokines Concentrations (pg/mL)	*p*	r
G-CSF	Fractalkine	<0.01	0.790
INFα2	GM-CSF	<0.01	0.820
sCD40L	EGF	<0.01	0.590
GM-CSF	MCP-1	<0.01	0.580

## Data Availability

The data presented in this study are available on request from the corresponding author. The data are not publicly available due to privacy reasons.

## Supplementary Materials

The following supporting information can be downloaded at: https://www.mdpi.com/article/10.3390/ijms25031565/s1.

## Abbreviations

**CCL11**-C-C motif chemokine 11, **CPT**—central point thickness, **CSF**—central subfield thickness, **CSII**—continuous subcutaneous insulin infusion, **CXCL8**–C-X-C ligand 8 motif, **EGF**—epidermal growth factor, **Flt-3 ligand**—fms-like tyrosine kinase 3, **G-CSF**–granulocytes colony-stimulating factor, **GM-CSF**—granulocytes-macrophages colony-stimulating factor, **GRO**—growth-regulating oncogene/**CXCL1**-C-X-C Motif Chemokine Ligand 1, **HbA1c**—glycated hemoglobin, **HDL-C**—high-density lipoprotein cholesterol, **IFN-α2**—interferon alpha-2, **IFN-γ**-interferon-gamma, **IL1ra**—interleukin 1 receptor antagonist, **IMT**—intima-media thickness, **IP-10**-interferon gamma-induced protein 10/**CXCL10**-C-X-C motif chemokine ligand 10, **LDL-C**—low-density lipoprotein cholestoerol, **MDC**—macrophage-derived chemokine/**CCL22**-C-C motif ligand, **MCP**-**1**-monocyte chemoattractant protein-1/**CCL2**-C-C motif ligand 2, **MCP-3**-monocyte-chemotactic protein 3/**CCL7**-C-C-motif ligand 7, **MDI**—multiple dose injections, **MIP-1α**—macrophage inflammatory protein-1 *alpha*, ***CCL3***-C-C—motif ligand 3, **MIP-1β**-macrophage inflammatory protein 1 beta/**CCL4**-C-C motif ligand 4, **T1D**—type 1 diabetes, **TC**—total cholesterol, **TDI**—total daily dose insulin, **TGF-α**—transforming growth factor alpha, **TNF-α**—tumor necrosis factor-alpha, **sCD40L**—soluble cluster of differentiation 40, **VEGF**—vascular endothelial growth factor.
