# Supplementary material for: The Complex Network of Cytokines and Chemokines in Pediatric Patients with Long-Standing Type 1 Diabetes
Source: Int J Mol Sci. 2024 Jan 26;25(3):1565. doi: 10.3390/ijms25031565 (PMC10855710; doi:10.3390/ijms25031565)
Supplement: Supplementary file 1 [file ijms-25-01565-s001.zip › ijms-2786409-supplementary.pdf]

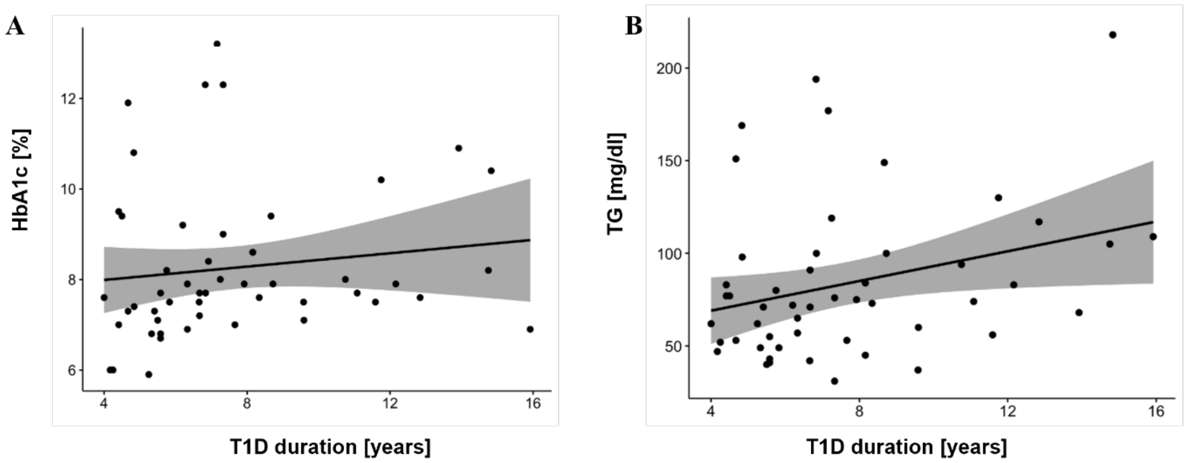

Figures S1A-B. The correlation between the T1D duration [years] and HbA1c level [%], and between the T1D duration [years] and TG level [mg/dl]. Correlations between measured parameters were analyzed using Spearman's rank correlation.

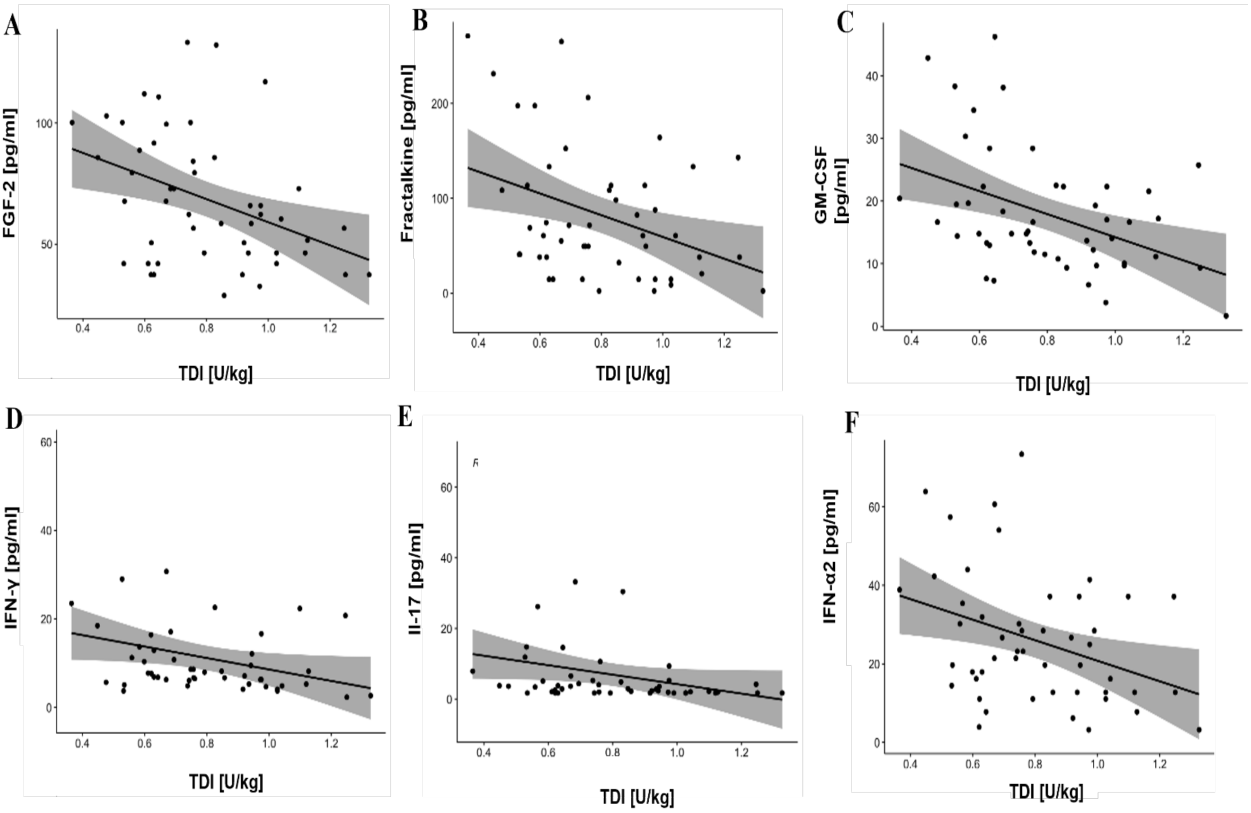

Figures S2A-F. Statistical significant correlations between biomarkers concentrations [pg/ml], and TDI [U/kg] in T1D patients. Logarithmic transformation of TDI was required.

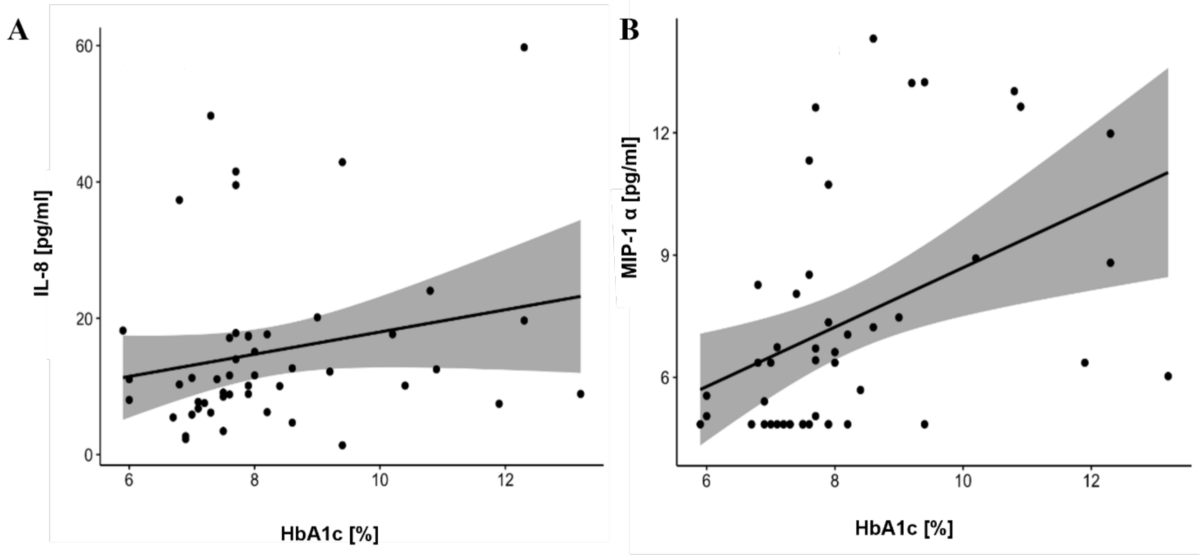

Figures S3A-B. Statistical significant correlations between cytokines concentrations [pg/ml] and HbA1c level [%] in T1D patients. Correlations between measured parameters were analyzed using Spearman's rank correlation.

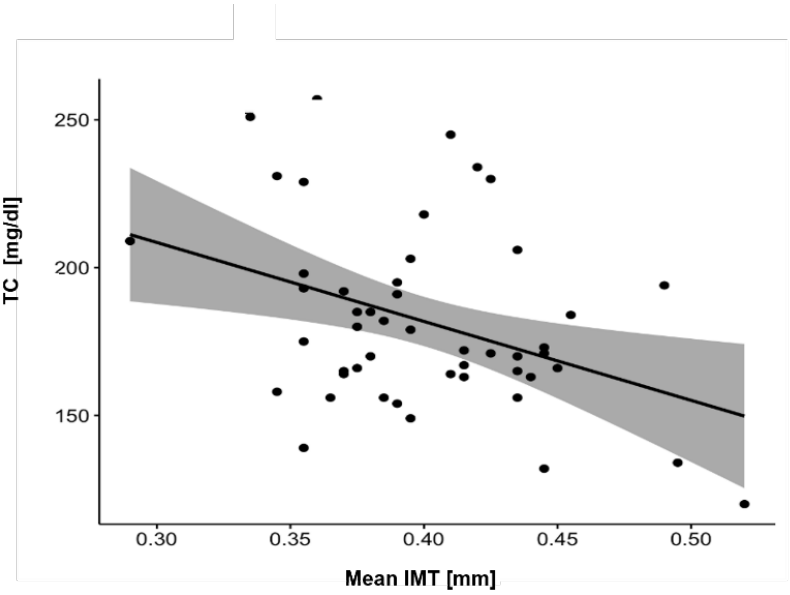

Figure S4. Statistical significant correlations between TC level [mg/dl] and mean carotid IMT [mm]. Correlations between measured parameters were analyzed using Spearman's rank correlation.

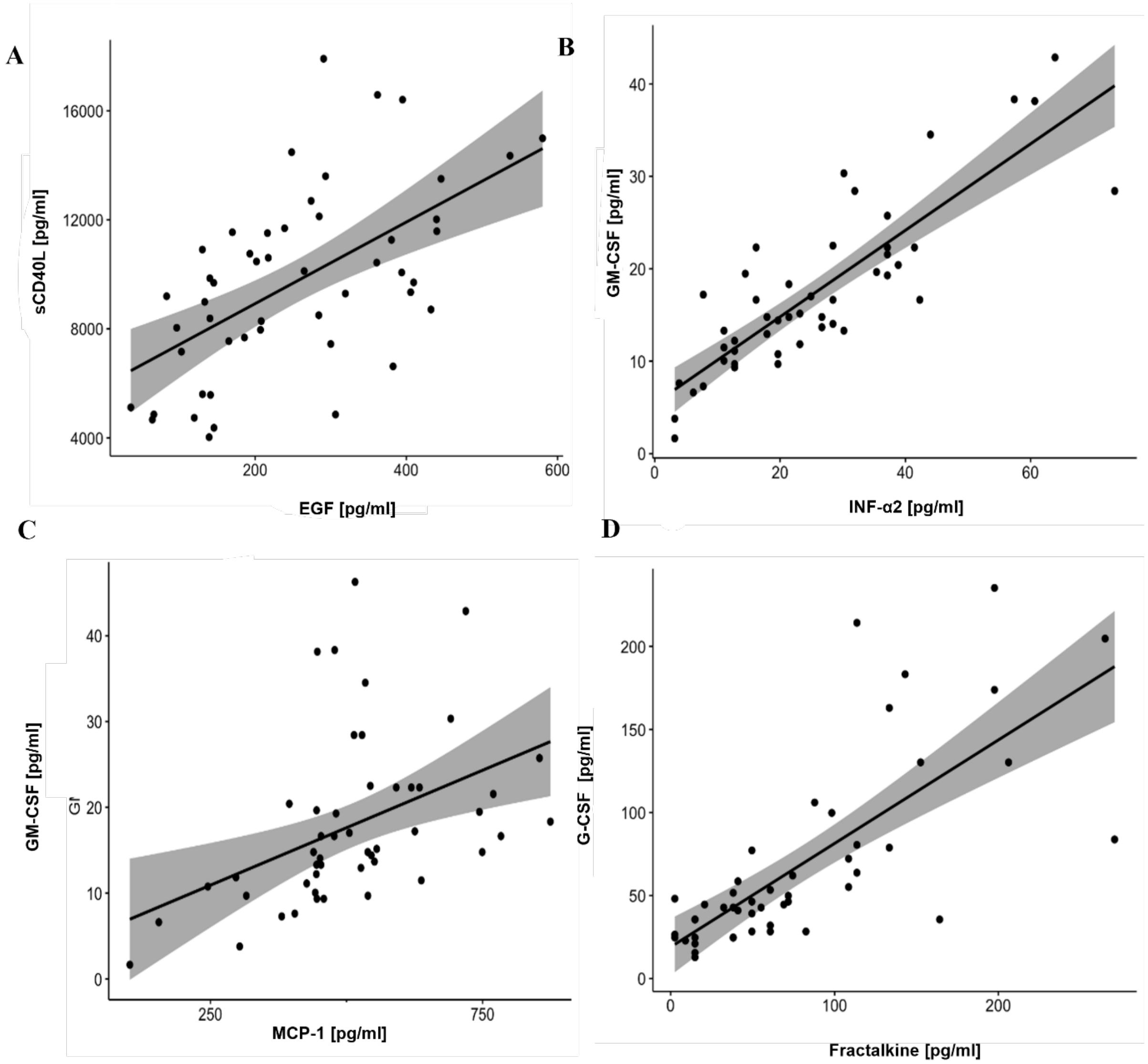

Figure S5A-D. Statistical significant correlations between biomarkers concentrations [pg/ml]. Correlations between measured parameters were analyzed using Spearman's rank correlation.

Table S1. Cytokines concentrations in T1D patients with HbA1c ≥ 8% and <8% in comparison to the HD group. Data are presented as mean values ± SD. F values and q values (adjusted p-values) were reported in the case of the ANOVA test.

**Cytokines concentrations (pg/ml)**	**T1D≥8%(n=20)**	**T1D<8%(n=32)**	**HD (n=39)**	**p-value**	**F**	**qT1D≥8%/ T1D<8%**	**qT1D≥8%/ HD**	**qT1D<8%/ HD**
EGF	283.7±147.7	233.3±117.6	162.6±83.9	<0.001	8.271	0.122	0.001	0.015
Eotaxin	152.7±83.0	115.5±44.2	102.2±33.0	0.003	6.307	**0.020**	0.002	0.279
MDC	1042.5±272.9	944.7±225.2	871.2±238.9	0.043	3.261	0.206	0.039	0.206
MCP-1	563.2±122.8	477.4±170.7	450.5±141.4	0.029	3.695	0.075	0.025	0.451
sCD40L	11129.3±3905.0	8897.3±2694.6	7645.8±2638.8	<0.001	8.839	**0.016**	<0.001	0.080
TNF-α	27.6±9.1	24.2±7.3	20.4±6.0	0.002	6.868	0.101	0.002	0.045
**× Cytokines concentrations (pg/ml)**	**T1D≥8% (n=20)**	**T1D<8% (n=32)**	**HD (n=39)**	**p-value**	**Chi-square**	**qT1D≥8%/ T1D<8%**	**qT1D≥8%/ HD**	**qT1D<8%/ HD**
MIP-1 α	7.5 (6.5)	5.1 (2.0)	5.1 (3.3)	0.011	11.285	**0.005**	0.006	0.878
TGF-α	7.2 (5.0)	6.0 (3.8)	3.7 (2.9)	<0.001	19.677	0.565	0.001	<0.001

× Data that failed to show normal distribution even following the log-transformation were analyzed using the Kruskal-Wallis test with post-hoc Wilcoxon test and are presented as medians with corresponding interquartile ranges (IQR).
